# Genome drafts of *Pseudogracilibacillus* spp. from soil and sediment of Mount Makiling, a dormant volcano in the Philippines

**DOI:** 10.1128/mra.00374-24

**Published:** 2024-07-05

**Authors:** Noel H. Tan Gana, Cernan P. Ruz, Edwin P. Alcantara, Maria Genaleen Q. Diaz, Eufrocinio C. Marfori, Lucille C. Villegas, Armi R. Creencia, Eldrin D. L. R. Arguelles, Neil H. Tan Gana, Rosario G. Monsalud, Rina B. Opulencia

**Affiliations:** 1 National Institute of Molecular Biology and Biotechnology, University of the Philippines Los Baños, College, Laguna, Philippines; 2 Institute of Biological Sciences, College of Arts and Sciences, University of the Philippines Los Baños, College, Laguna, Philippines; 3 Department of Biochemistry and Molecular Biology, College of Medicine, University of the Philippines Manila, Manila, Philippines; 4 Department of Arts and Sciences, City College of Calamba, Calamba, Laguna, Philippines; DOE Joint Genome Institute, Berkeley, California, USA

**Keywords:** Bacillaceae, Illumina, taxonomy, bacterial phylogeny, genomes

## Abstract

We present the draft whole-genome sequences of *Pseudogracilibacillus* spp. isolated from the soils and sediments of Sipit Creek located at Mount Makiling, a dormant volcano in the southern part of Luzon Island, Philippines. This P*seudogracilibacillus* spp. genome report extends the body of knowledge on a lesser-known genus of Bacillaceae.

## ANNOUNCEMENT

Discoveries and genome-wide understanding of *Pseudogracilibacillus* and related genera are key to resolving the taxonomic ambiguities of the polyphyletic and highly functional bacterial family Bacillaceae. This submission describes possibly three distinct new strains of *Pseudogracilibacillus* spp. with genome sizes of 2,900,005 bp (SO10305), 4,652,103 bp (SO30301A), and 4,065,421 bp (SE30717A). The genome coverages were 249.96 (SO10305), 281.23 (SO30301A), and 268.98 (SE30717A). The *Pseudogracilibacillus* SO10305, SO30301A, and SE30717A genome annotations, respectively, predicted 2,870, 4,562, and 3,974 total genes; 2,820, 4,512, and 3,922 total CDS; and 2,790, 4,320, and 3,881 total CDS with proteins, which concurred to *Pseudogracilibacillus* spp. associated GenBank Data. The annotation also predicted 50 (SO1030550 and SO30301A) and 52 (SE30717A) total RNA genes, 2 rRNAs (all strains), 5 ncRNAs (all strains), and 43 (SO10305 and SO30301A) and 45 (SE30717A) tRNAs.

The *Pseudogracilibacillus* spp. were isolated from Sipit Creek sediments and adjacent soils sampled within a 5 × 5-m plot transect at the Mt. Makiling Forest Reserve, Philippines (14.102713, 121.201460) on 20 September 2019. Spread plating of samples in modified peptone yeast extract glucose agar supplemented with 10 ppm cycloheximide incubated at 30°C for 30 days was performed to obtain pure cultures (1). The isolates were initially identified through *16S rRNA* capillary sequencing (AB Systems 3730xl) using 27F-AGAGTTTGATCCTGGCTCAG, 1429R-TACGGYTACCTTGTACGACTT, 518F-CCAGCAGCCGCGGTAATACG, and 800R-TACCAGGGTATCTAATCC primers. The *16S rRNA* gene sequences were most similar to *Pseudogracilibacillus auburnensis* P-207(T) (2) at 95.3% and 97.43% for SO10305 and SE30717A, respectively, and *P. endophyticus* DT02-17 (T) for SO30301A at 97.5%.

Purified total genomic DNAs isolated using the NucleoSpin Microbial DNA Isolation Kit (Machery-Nagel, Germany) were sent to Macrogen Inc. (Seoul, South Korea). TruSeq Nano DNA (350) was used to generate the libraries, and its sequencing in the Illumina NextSeq 1000 platform yielded 101-bp paired-end reads. Trim Galore version 0.6.7 (3) and FastQC (4) were used to yield Q30 reads with ≥90.9% average quality. SPAdes V3.13.0 (5), Pilon version 1.23 (6), and UniCycler 0.5 (7) were sequentially used for genome *de novo* assembly, polishing, and final assembly implementation. Prokka v. V1.14.5 (8) was utilized for genome annotation and coding sequence identification. EggNOG mapper V-2.1.3 (9) and eggnog Database 5 (http://eggnog5.embl.de/) were used to map proteins into different functional categories. CheckM V1.2.1 (10) was used to determine genome completeness. MGCplotter (11)-generated genome plots are available on FigShare. All utilized software employed default parameters. Table 1 contains the *Pseudogracilibacillus* spp. detailed genomic information in this report.

**TABLE 1:** Genome feature data of *Pseudogracilibacillus* spp. strains

Genome features	*Pseudogracilibacillus* sp. SO10305	*Pseudogracilibacillus* sp. SO30301A	*Pseudogracilibacillus* sp. SE30717A
Totality of bases (bp)	910,874,358	1,024,856,494	980,209,848
Number of raw reads (bp)	9,018,558	10,147,094	9,705,048
Genome reads coverage^*a*^	249.96	281.23	268.98
Quality of reads after trimming (Q20/Q30 %)	94.8/90.9	94.8/91.3	95.1/91.8
Genome size, bp	2,900,005	4,652,103	4,065,421
G + C content (%)	35.03	35.4	35.09
N_50_ contig length, bp	128,717	69,890	355,079
L_50_ contig	7	19	5
Number of contigs^*b*^	42	140	25
Genome completeness (CheckM analysis v1.2.2)^*c*^	96.95%	97.15%	97.44%
Genome contamination (CheckM analysis v1.2.2)^*c*^	0.05%	2.15%	1.51%
Number of genes (total)^*b*^	2,870	4,562	3,974
Number of CDS (total)^*b*^	2,820	4,512	3,922
Number of genes (coding)^*b*^	2,790	4,320	3,881
Number of CDS (with protein)^*b*^	2,790	4,320	3,881
Number of genes (RNA)^*b*^	50	50	52
rRNAs^*b*^	1, 1 (16S, 23S)	1, 1 (16S, 23S)	1, 1 (16S, 23S)
tRNAs^*b*^	43	43	45
ncRNAs^*b*^	5	5	5
SRA accession	SRR27027419	SRR27027420	SRR27027418
BioSample accession	SAMN38440224	SAMN38440223	SAMN38440225
GenBank accession	JAXHDE000000000	JAXHDD000000000	JAXHDF000000000

^*a*^ The reference genome used was *Pseudogracilibacillus auburnensis* with 3644.147 bp size.

^*b*^ Annotation methodology used Prokka v. V1.14.5.

^*c*^ Calculated by the Prokaryotic Genome Annotation Pipeline with the Bacillaceae CheckM marker.

The FAST average nucleotide identity (ANI; https://github.com/ParBLiSS/FastANI) (12) analysis of *Pseudogracilibacillus* spp. showed their ANI below 80%, implying the three isolates were different species. Their similarity to *Pseudogracilibacillus auburnensis* P-207(T) is below the 70% threshold. Further digital DNA-DNA hybridization (13) confirmed the genome-to-genome distances among strains SO10305, SO30301A, and SE30717A, and available type strains were below 70%, the distinguishing threshold for bacterial species. Hence, we present three *Pseudogracilibacillus* spp. isolates originating from the volcanic soils and sediments of Mt. Makiling, Luzon Island, the Philippines.

## Data Availability

This Whole-Genome Shotgun project has been deposited in GenBank under accession numbers JAXHDE000000000 (SO10305), JAXHDD000000000 (SO30301A), and JAXHDF000000000 (SE30717A). Version (01) described in this paper has accession numbers JAXHDE010000000 and sequences JAXHDE010000001-JAXHDE010000042 (SO10305), JAXHDD010000000 and sequences JAXHDD010000001-JAXHDD010000140 (SO30301A), JAXHDF010000000 and sequences JAXHDF010000001-JAXHDF010000025 (SE30717A). The BioProject accession PRJNA1045277 includes the BioSample and SRA accession numbers hyperlinked in Table 1.
